# The Ladies

**Published:** 1902-06-15

**Authors:** B. Le Gro


### THE LADIES.

A TOAST BY DR. B. LE GRO,

At the Banquet to the Southwestern Michigan Dental Society at Buchanan, April 8, 1902.

How can I toast them? The ladies. These miracles of divine contradiction. How can I successfully speak for myself on "The Ladies" in a bunch, when I have been unable to successfully speak for myself to just one, traveling alone. I've been energetic along that line too, keeping ever in mind the old motto "If at first you don't succeed, try, try again." But really the subject is a hard one. The ladies are difficult of approach; I'm bashful. I might resort to telling stories, but even that has its dangers in this day and age when a story travels so fast—especially among the ladies. A new story to me might be an old story to you. It has been said that "A woman is like your shadow; follow her, she flies; fly from her, she follows." I have tried both and up to the present time it has been a case of fly all of the time without any accompanying following, but then we disappointed bachelors can seek consolation in the saying of that sage, who presumably became a benedict—"Of all men Adam was the happiest; he had no mother-in-law." You generally get one in every package nowadays.

Woman is the puzzle of the ages—the accepted symbol of the unknown—the approved type of the mysterious. She is an insoluble riddle. The things men have said about her would fill a book as long as the bell-rope to a freight train and are about as far apart as is the caboose from the engine. Amiel calls woman "the monster incomprehensible." Lessing thought God meant to make woman his masterpiece, and Milton deemed her a fair defect of nature; Shakespeare calls her another name for frailty and Holmes thinks her the "Messiah of a new faith." All agree that flattered ladies are indulgent, and Balzac says "A man must be a fool who does not succeed in making a woman believe that which flatters her." In other words Balzac gives testimony in favor of the use, to the fair sex, of what is commonly known as "hot air." Balzac would be a success in my position to-night. Some one has said that "There will be so many more women in heaven than men, that any marriage, except of the Mormon kind, will be impossible," this fact, if it be one, certainly furnishes a goal for some of us. Henceforth I am a Christian.

But seriously, the ladies are the greatest inspiration we have; the inspiration of poet and painter; the theme of Homer's Iliad, the greatest poem ever written—"Helen of Troy;" the inspiration of more artists than any other subject, "The Madonna," the highest type of motherhood; but why continue the list that begins with the "Isles of Greece," where "Burning Sappho loved and sang," and continues uninterrupted down to the present day. Why, I venture the assertion—there is not a man present here to-night who has not at some time in his life been poetically inspired by some woman's presence. Now you would never suspect our toastmaster of any esthetic tendencies; but he remembers that little girl with the flaxen hair done up in pigtails, whose picture he used to draw when the teacher wasn't looking and under which he wrote the emblematic verse, "If you love me as I love you, no life can cut our love in two," and some of you remember well the morning you sided up to a little brown-haired girl on the way to school and handed her a rose and a note with this message, "The rose is red, violets blue; Sugar is sweet and so are you." Of course you remember it. We all do. We have all been there.

Way back in the beginning of history we see our mother Eve arrayed in her primeval garb, start the ball of human history rolling by doing the first act worthy ot record. Look at the roll since then, the Queen of Sheba, Cleopatra, Joan of Arc, Queen Victoria, Carrie Nation, all mighty conquerors and world-renowned leaders of history. And to-day by every fireside where she sits, mending stockings and patching small trousers and coats, while she sings lullabies over the cradle, she in this sphere, her true one, is writing indelibly, the future of the nation. It is not a poet's dream that "the hand that rocks the cradle is the hand that rules the world."

The Almighty knew what he was talking about when he looked at the creature he had made and said, "It is not well for man to be alone, I will make a helpmate for him"—for such she has ever been, steadfast and true, and when all others fail, comforting and cheerful when sick and despondent, and by her womanly purity and strength of character, lifting us up to a higher and nobler existence. And when I say to-night: "The ladies, may God bless them, and may they live long and be happy; and for the good of the human race, when they cease to exist, may there be no more men upon the earth."

Every man in this audience who has known the tender affection and self-sacrificing love of a friend, sweetheart, sister, wife or mother, will be proud to say "them's my sentiments."

